# Recent Advances in the Biosynthesis of Natural Sugar Substitutes in Yeast

**DOI:** 10.3390/jof9090907

**Published:** 2023-09-07

**Authors:** Jian Li, Honghao Li, Huayi Liu, Yunzi Luo

**Affiliations:** 1 Frontiers Science Center for Synthetic Biology and Key Laboratory of Systems Bioengineering (Ministry of Education), School of Chemical Engineering and Technology, Tianjin University, Tianjin 300072, China; li_jian0604@163.com (J.L.); honghaoli@tju.edu.cn (H.L.); huayi0814@126.com (H.L.); 2 Georgia Tech Shenzhen Institute, Tianjin University, Tangxing Road 133, Nanshan District, Shenzhen 518071, China

**Keywords:** natural sugar substitutes, microbial cell factories, metabolic engineering, synthetic biology, yeast

## Abstract

Natural sugar substitutes are safe, stable, and nearly calorie-free. Thus, they are gradually replacing the traditional high-calorie and artificial sweeteners in the food industry. Currently, the majority of natural sugar substitutes are extracted from plants, which often requires high levels of energy and causes environmental pollution. Recently, biosynthesis via engineered microbial cell factories has emerged as a green alternative for producing natural sugar substitutes. In this review, recent advances in the biosynthesis of natural sugar substitutes in yeasts are summarized. The metabolic engineering approaches reported for the biosynthesis of oligosaccharides, sugar alcohols, glycosides, and rare monosaccharides in various yeast strains are described. Meanwhile, some unresolved challenges in the bioproduction of natural sugar substitutes in yeast are discussed to offer guidance for future engineering.

## 1. Introduction

The recent worldwide increase in the incidence of chronic diseases, such as type 2 diabetes, hyperlipidemia, hypertension, and obesity, has become a leading cause of death [1]. Evidence from both observational and clinical studies has revealed that the excess intake of unhealthy foods containing high amounts of sugar, fat, and salt is the main cause of the incidence of such chronic diseases [1,2]. Reducing the excess intake of high-calorie sugars can lower the risks of these diseases [3]. The World Health Organization (WHO) recommends that individuals limit their dietary sugar intake to less than 10% of their total daily energy intake [4]. However, sweetness is a basic taste favored by humans worldwide. Consequently, the daily sugar intake of humans frequently exceeds the recommended limit. Therefore, researchers have turned their focus to sugar substitutes to reduce the intake of calories and free sugars. Although low-calorie artificial sweeteners, such as saccharin, aspartame, cyclamate, and sucralose, have been used since the 1800s, the risks associated with them have always been a hot topic in the scientific community and in society [5].

Natural sugar substitutes are usually safe, sufficiently sweet, and low in calories [3,6]. To meet the increasing market demand for artificial sweeteners, reliable manufacturing methods for natural sugar-substitute production are essential. Currently, plant extraction and chemical synthesis are the main production processes, which are often unsustainable, require high energy, and cause environmental damage [7]. Biosynthesis using microbial fermentation is usually safe and environmentally friendly. The advances in microbial synthetic biology in the past decade have facilitated the sustainable production of a wide array of biological products, such as biopharmaceuticals, biofuels, and biomaterials [8,9,10,11]. Thus, construction of microbial cell factories via synthetic biology tools to manufacture natural sugar substitutes is a promising alternative to chemical synthesis.

Yeast is an interesting microbe for natural product biosynthesis, as it is safe and easy to handle and its genetic tools are widely available [12,13]. Moreover, researchers have successfully demonstrated the production of natural sugar substitutes in yeast [14,15,16]. Numerous synthetic biology and metabolic engineering strategies, such as increasing the availability of substrates, alleviating the catabolite repression, and optimizing the redox balance, have been previously reported as prompting natural sugar substitute production in yeast. In this review, we provide an overview of the recent instances of progress made in the biosynthesis of different classes of natural sugar substitutes in yeast, especially oligosaccharides, sugar alcohols, glycosides, and rare monosaccharides.

## 2. Biosynthesis of Oligosaccharides

Oligosaccharides consist of 2–10 monosaccharides of the same or different types. The oligosaccharides commonly biosynthesized in microbes are 2′-fucosyllactose and trehalose [17,18]. These compounds are found to benefit immunity and regulate the gut microbiota [19]. In this section, we will review metabolic engineering efforts in producing oligosaccharides using yeasts.

### 2.1. 2′-Fucosyllactose (2′-FL)

2′-Fucosyllactose (2′-FL) is one of the oligosaccharides found in human milk (human milk oligosaccharide, HMO), which plays a crucial role in protecting infants against immunological diseases [20,21]. Hence, 2′-FL is not only a sugar substitute but also a milk analog [22]. 2′-FL has a structure of *l*-fucose connected to lactose on the end of galactose, and currently is mainly synthesized by chemical or enzymatic reactions [23]. However, its process of chemical synthesis requires environmentally toxic compound selenides and other organic solvents [24]. The enzymatic transformation of 2′-FL is catalyzed by the α-1,2-fucosyltransferase (FutC), with GDP-*l*-fucose and lactose as substrates [23]. The high cost of the expensive substrate and purified enzyme hinders its scaling-up in industry. Therefore, researchers have started the biosynthesis of 2′-FL from low-cost substrates in model microbes, particularly yeast, in the past few years.

**Figure 1:**
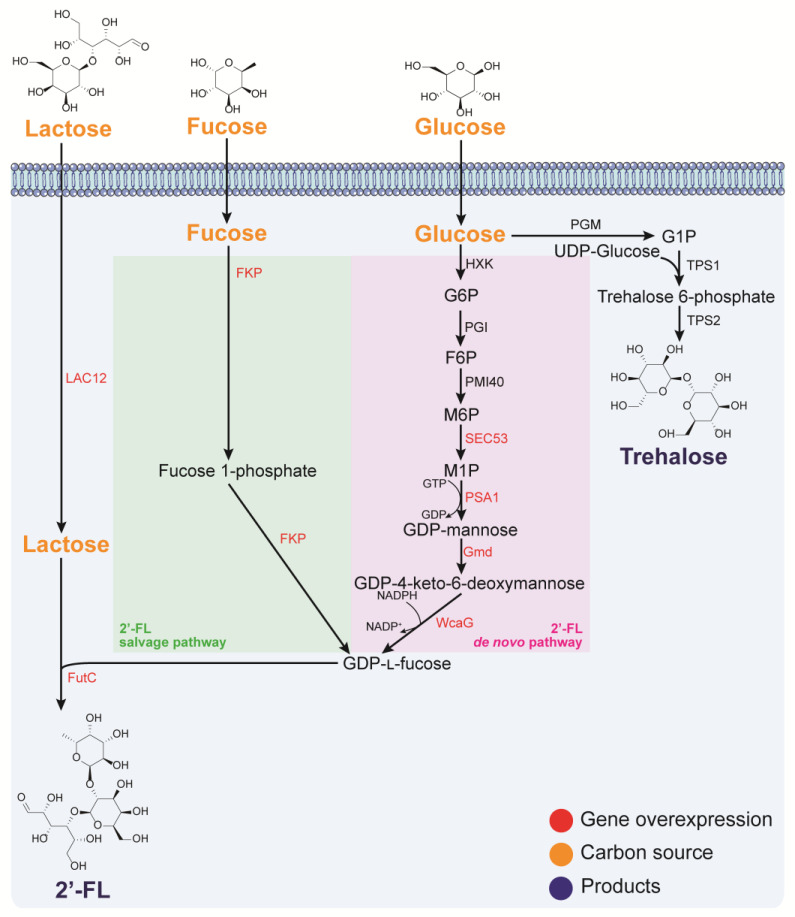
Biosynthetic pathway of 2′-fucosyllactose and trehalose in yeasts. LAC12: lactose permease from *K. lactis*; FKP: fucokinase/GDP-*l*-fucose pyrophosphorylase; FutC: α-1,2-fucosyltransferase; PGI: phosphoglucoisomerase; PMI40: mannose 6-phosphate isomerase; SEC53: phosphomannomutase; PSA1: αD-mannose 1-phosphate guanylyltransferase; Gmd: GDP-mannose 6-dehydrogenase; WcaG: GDP-*l*-fucose synthase; PGM: phosphoglucomutase; TPS1: trehalose phosphate synthase; TPS2: trehalose 6-phosphate phosphatase; GTP: guanosine 5′-triphosphate; GDP: guanosine 5′-diphosphate; UDP-Glucose: uracil diphosphate glucose.

In vivo biosynthesis of 2′-FL requires the condensation of 1 part molar lactose and 1 part molar GDP-*l*-fucose, catalyzed by FutC. The formation of GDP-*l*-fucose can be achieved via two routes, the de novo and salvage pathways (Figure 1) [17]. The de novo pathway comprises three enzymatic steps starting from fructose 6-phosphate to GDP-mannose, catalyzed by PMI40, SEC53, and PSA1 originated from yeast, respectively. Then, GDP-mannose is converted to GDP-*l*-fucose via two enzymatic steps catalyzed by GDP-mannose-4,6-dehydratase (Gmd) and GDP-*l*-fucose synthase (WcaG) from *Escherichia coli* [25]. In contrast, the salvage pathway is much simpler, as it involves only two enzymes or just one bifunctional enzyme (Fkp) that facilitate phosphorylation and GDP-transfer [26].

Yeast species are excellent platforms for 2′-FL biosynthesis, as yeasts usually produce a sufficient amount of GDP-mannose to synthesize glucan in its cytoderm, while GDP-mannose is also the precursor of GDP-*l*-fucose [22,27,28,29,30,31]. For *Saccharomyces cerevisiae* or *Yarrowia lipolytica*, lactose cannot be taken as the carbon source, indicating that intracellular lactose could probably be directed into the biosynthesis of 2′-FL. Previously, lactose permeases (Lac12s) in the *Kluyveromyces* genus were reported to transport lactose into the cytoplasm; therefore, heterologous expression of Lac12s in *S. cerevisiae* or *Y. lipolytica* is a common strategy for importing lactose [32]. An engineered *S. cerevisiae* strain with the heterologous expression of *Kluyveromyces lactis* Lac12, *Bacteroides fragilis* Fkp, and *Helicobacter pylori* FucT2 (isoenzyme of FutC) produced 92 mg/L of 2′-FL in a shake flask containing 20 g/L glucose, 2 g/L lactose, and 2 g/L *l*-fucose in 48 h, and 503 mg/L of 2′-FL in a 120-h batch fermentation was achieved (Table 1) [22]. In another study, 0.51 g/L of 2′-FL was produced via the de novo pathway using a medium containing 40 g/L glucose and 3 g/L lactose. This titer could be increased, as the export of 2′-FL was a limitation in these strains [27]. To solve this issue, researchers analyzed a series of potential 2′-FL transporters and identified that *Neurospora crassa* CDT2 was the best-performing candidate. The ratio between extracellular and intracellular 2′-FL in the engineered strain was 1.8-fold more than that of the control strain [28]. This study improved the transportation of 2′-FL, but the upstream carbon source utilization issue also needs to be considered. The predominantly fermentative nature of glucose metabolism in *S. cerevisiae* might limit 2′-FL productivity. Xylose was selected as an alternative carbon source to balance strain growth and production, releasing *S. cerevisiae* from the Crabtree effect, which would repress respiration and facilitate ethanol production under glucose utilization [33]. By heterologous expression of the *Scheffersomyces stipitis* XYL1/2/3 genes in *S. cerevisiae*, the engineered strain could produce 2′-FL utilizing xylose as a carbon source. Finally, this strain produced 1.6 g/L of extracellular 2′-FL in a shake flask and 25.5 g/L of 2′-FL (4.5 g/L of extracellular 2′-FL) from fed-batch fermentation using xylose and lactose as substrates [29]. 

As the substrates are saccharides, dynamic regulation between growth and production is essential for the 2′-FL production titer [46]. The inducible promoters *P_gal1_* and *P_gal10_* were employed to regulate the expression of the *Sec53* and *Psa1* genes in the endogenous GDP-mannose pathway, and a highly efficient FutC from *Bacillus cereus* (FutBc) was selected. This strain produced 3.45 g/L (72 h, shake flask) and 19.56 g/L (fed-batch) of 2′-FL [30]. Moreover, dynamic regulation based on quorum-sensing activated the inducible promoters *P_gal1_* and *P_gal10_* by amplifying the response output of a galactose-inducing system. As a result, the expression of heterologous genes WcaG and FutBc (by *P_gal1_*), as well as Gmd (by *P_gal10_*), was enhanced dynamically. The 2′-FL titer increased to 3.37 g/L (48 h, shake flask) and 25.73 g/L (fed-batch) [31]. The de novo biosynthetic pathway of 2′-FL in yeast chassis exhibited promising results. However, the consumption of GDP-mannose and the synthesis of cytoderm in yeast should be regulated for optimal yields of 2′-FL. A study assessing *S. cerevisiae*, which attempted to engineer the cofactors NADPH and GTP to release the limitation on the metabolic flux of GDP-*l*-fucose, was carried out, but no significant improvement in the 2′-FL yield was observed [30]. Hence, other familiar strategies for increasing the cofactors’ supply, including overexpressing related genes or mining other NADP^+^-dependent enzymes, merit further evaluation. In addition, more emphasis can be focused on the dynamic regulation between cell growth and 2′-FL biosynthesis.

### 2.2. Trehalose

Trehalose is 0.45 times less sweet than sucrose [47], and is a non-reductive disaccharide containing two molecules of glucose with a α-(1→1)-glycosidic form bond. It can be used in the food industry as a preservative or desiccant, as its structure is stable, and it can easily form bonds with water. Trehalose is also a distress indicator, as it is produced to protect cells against harsh environmental conditions. However, the current synthesis methods for trehalose—chemical synthesis and extraction from yeast—are expensive, which limits its commercialization.

Two groups of enzymes (MTS-MTH and TreS) are commonly used in the industrial production of trehalose [3]. Maltooligosyltrehalose synthase (MTS) and maltooligosyltrehalose trehalohydrolase (MTH) produce trehalose from oligosaccharides with a α-glycosidic bond (starch, maltodextrin, etc.). The MTS-MTH method was the first enzymatic approach to produce trehalose. However, MTS also catalyzes the isomerization of amylose with more than three saccharides, resulting several by-products [48]. Trehalose synthase (TreS), which converts maltose to trehalose directly in one step, was applied in the industrial production of trehalose to bypass this issue [48,49]. Meanwhile, fusion expression of *Picrophilus torridus* TreS and the *Y. lipolytica* anchor protein Pir1 facilitated the production of trehalose outside the cells, prompting the trehalose titer to 219 g/L with 300 g/L of maltose substrate [18,34].

The trehalose biosynthetic pathway in yeast includes the enzyme complex formed by Tps1, Tps2, Tps3, and Tsl1 (Figure 1). Tps1 and Tps2 are trehalose phosphate synthase (TPS) and trehalose 6-phosphate phosphatase (TPP), respectively, while Tps3 and Tsl1 are regulatory proteins. However, the intermediate trehalose 6-phosphate inhibits the expression of Tps1. The expression of the Tps1-Tps2-Tps3-Tsl1 complex is induced under stress conditions [50]. Therefore, researchers increased the intracellular trehalose concentration by regulating stress-resistant related genes in yeast [51,52]. However, to date, a high titer of trehalose has not been achieved with an engineered yeast strain through the endogenous pathway. This is attributed to the complicated metabolic regulation around trehalose biosynthesis, strong growth inhibition in the presence of a high titer, and absence of a highly efficient transporter. Hence, further investigations on the transcriptomics, proteomics, and metabolomics of the trehalose metabolic network in yeast are needed.

## 3. Biosynthesis of Sugar Alcohols

Sugar alcohols, known as saccharide derivatives, are noncyclic hydrogenated carbohydrates formed by the reduction of the aldehydes or ketones in sugars to hydroxyl groups [7]. Yeasts can produce mannitol, erythritol, xylitol, sorbitol, and threitol naturally or via genetic engineering [53,54]. Extensive literature reviews on the biosynthesis of sugar alcohols have been previously reported, giving a nice scheme for the strategies applied so far [7,42,44]. Therefore, in this review, we only focus on the recent advances in the yeast-biosynthesis techniques of mannitol, erythritol, and threitol and their industrial-scale production potential.

### 3.1. Mannitol

Mannitol is a six-carbon sugar alcohol found in various plants, algae, and the mycelia of various fungi and is one of the main carbohydrates in mushrooms. It is widely used in the food, pharmaceutical, and medical industries, as it is low-calorie and exhibits non-cariogenic properties [55]. A report by Market Monitor Co., Ltd (Changsha, China). revealed that the global mannitol market size is estimated to grow to USD 352.35 million by 2028. Industrial production of mannitol involves the hydrogenation of fructose at high temperature and pressure, which requires high energy and causes environmental damages. The microbial synthesis of mannitol could become a promising alternative as microorganisms produce mannitol via the fermentation of glucose or fructose [7,54]. Metabolic engineering is required to optimize mannitol yields in microorganisms.

**Figure 2:**
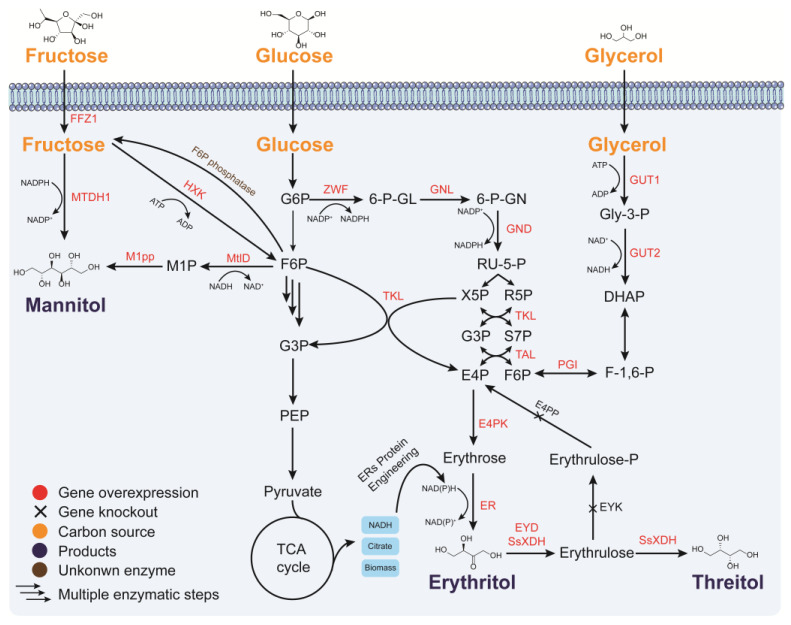
Schematic of the metabolic pathways and key enzymes for the production of sugar alcohols in yeasts. ZWF: glucose-6-phosphate 1-dehydrogenase; GNL: 6-phosphogluconolactonase; GND: 6-phosphogluconate dehydrogenase; TKL: transketolase; TAL: transaldolase; PGI: phosphoglucose isomerase; E4PK: erythrose-4-phosphate kinase; ER: erythrose reductase; EYD: erythrulose dehydrogenase; SsXDH: xylitol dehydrogenase gene from *Scheffersomyces stipitis*; EYK: erythrulose kinase; E4PP: erythrose 4-phosphate phosphatase; GUT1: glycerol kinase; GUT2: glycerol 3-phosphate dehydrogenase; HXK: hexokinase; MtlD: mannitol-1-phosphate dehydrogenase; M1pp: mannitol-1-phosphate phosphatase; FFZ1: fructose transporter; MTDH1: mannitol dehydrogenase; E4P: erythrose-4-phosphate; Gly-3-P: glyceraldehyde-3-P; DHAP: dihydroxyacetone phosphate.

Mannitol is biosynthesized via two different pathways (Figure 2): (i) the NADH-dependent conversion of fructose-6-phosphate (fructose-6-P) to mannitol 1-phosphate (mannitol-1P), followed by dephosphorylation to yield mannitol; and (ii) the direct conversion of fructose to mannitol through an NADPH-dependent mannitol dehydrogenase (MTDH1) [54]. Yeasts such as *S. cerevisiae*, *Candida magnolias*, and *Y. lipolytica* can produce mannitol by fermentation. The metabolic engineering strategy for mannitol production primarily focuses on enhancing the efficiency of NADH regeneration [53]. MtlD from *E. coli* is a bacterial NAD^+^-dependent mannitol-1-phosphate dehydrogenase that can transform fructose-6P and mannitol-1P into each other [56]. In modified *S. cerevisiae* strains, this enzyme enables the conversion of fructose-6P to mannitol-1P, followed by dephosphorylation by mannitol-1-phosphate phosphatase (M1pp) to produce mannitol while regenerating NAD^+^ [35]. Therefore, mannitol is produced under anaerobic conditions by a glycerol-defective mutant of *S. cerevisiae* expressing the MtlD gene. However, the mannitol yield (only 0.12 g/g glucose) is low. *Candida parapsilosis* SK26.001, isolated from sugarcane juice, can produce 97.1 g/L of mannitol in a 120-h fed-batch fermentation from high concentrations of glucose [37]. Moreover, Ca^2+^ and Cu^2+^ supplements enhanced mannitol production in *Candida albicans*. Ca^2+^ decreases the amount of intracellular mannitol by changing cell permeability, whereas Cu^2+^ increases mannitol dehydrogenase activity to afford a higher amount of mannitol. The mannitol titer was 223 g/L with a yield of 0.87 g/g fructose in the presence of Ca^2+^ and Cu^2+^ supplements, representing a 35% increase compared to the parent *C. magnoliae* HH-01 strain [38]. These studies demonstrate the potential of yeast as chassis for industrial-level mannitol production.

### 3.2. Erythritol

Erythritol is a four-carbon polyol that occurs naturally in algae, fungi, fruit, and fermented food [15]. It is a zero-calorie sweetener widely used in beverages, foods, and pharmaceuticals [57]. It is synthesized via the pentose phosphate pathway (PPP), starting from erythrose-4-phosphate (E4P). E4P is dephosphorylated to erythrose by erythrose 4-phosphate phosphatase (E4PP) and one molecule of NAD(P)H. Erythrose is then reduced to erythritol by erythrose reductase (ER). The process of biosynthesis relies on the microorganism’s capability of producing high yields of erythritol with least glycerol formation. Therefore, metabolic engineering strategies in the past decade have largely been focused on enhancing strain productivity, blocking erythritol degradation pathways, and optimizing the utilization of inexpensive substrates [15,39,43,58,59].

The current large-scale industrial production of erythritol is mainly achieved through the microbial fermentation of osmolytic yeasts such as *Y. lipolytica*, *Pseudozyma tsukubaensis*, and *Moniliella.* These yeasts are favored for commercial erythritol production due to their high yields and utilization of abundant, inexpensive, and recyclable substrates. *Y. lipolytica* can produce erythritol from glucose, fructose, mannose, and glycerol media [15]. Researchers improved erythritol production by screening generating mutants or optimizing the medium or culture conditions [40,60,61]. The mutant *Y. lipolytica* MK1 was obtained by UV mutagenesis, and the C:N ratio in the culture medium was optimized to achieve an erythritol titer of 113.1 g/L [60,61]. A sensor-regulatory system based on the erythritol-responsive transcription factor EryD was constructed to screen the mutant strain library. Taking advantage of high-throughput screening, a strain with an erythritol titer of 148 g/L was rapidly identified within a week [40]. This work demonstrably provides us a strategy for rapidly improving strain performance and engineering efficient microbial cell factories for industrial applications. In addition, improving the heat tolerance of *Y. lipolytica* could reduce the cost of both cooling the bioreactor and erythritol purification. The overexpression of the ubiquitin ligase encoded by RSP5 derived from *S. cerevisiae* improved the heat resistance [62]. By heterologous expression of the *S. cerevisiae* RSP5 genes in *Y. lipolytica*, the engineered strain could grow well at 35 °C and synthesize erythritol at 33 °C, which would reduce the cooling cost [41]. To further improve the heat resistance and production performance of *Y. lipolytica*, thirty potential heat-resistant genes with different functions were screened and tested in different thermotolerant strains. Eight proteins improved the growth of *Y. lipolytica* and the production of erythritol at 35 °C [63].

Metabolic engineering efforts to improve *Y. lipolytica* productivity have largely been focused on (i) tuning the flux of the precursor pathway, (ii) enhancing the metabolic activity of NAD(P)H, (iii) reducing by-product production, and (iv) optimizing the utilization of low-value substrates; consequently, erythritol yield and titer have been substantially improved [15,61,62]. In particular, overexpression of GUT1, GUT2, and TKL1 and the knocking out of EYD1 enabled the utilization of crude glycerol to produce 150 g/L erythritol with a yield of 0.62 g/g total substrate and a productivity of 1.25 g/L/h [15]. Moreover, the overexpression of the genes encoding glycerol kinase (GK) and transketolase (TKL) increased erythritol production in *Y. lipolytica* [64,65]. The heterologous expression of sugar alcohol phosphatase (PYP) combined with GK and TKL overexpression strains increased the yield of erythritol to 58.8 g/L during fed-batch growth in shake flasks, and the glycerol utilization rate by 2.5-fold [66]. The NADPH-dependent erythrose reductase (ER) in *Y. lipolytica* catalyzes the reduction of erythrose to erythritol. Constitutive expression of the newly isolated two erythrose reductase genes (ER10 or ER25), glucose-6-phosphate dehydrogenase gene (ZWF1), and 6-phosphogluconate dehydrogenase gene (GND1), led to a 23.5% higher erythritol yield and 50% higher productivity compared to the wild-type strain. The highest erythritol production titer of 190 g/L was achieved in a baffled-flask fermentation using glucose as feedstock. The higher erythritol production of the recombinant strain that overexpressed ZWF1 and GND1 could be attributed to the higher intracellular NADPH metabolic activity. This suggests that intracellular NADPH levels play a crucial role in the biosynthesis of erythritol.

*P. tsukubaensis* strains can also synthesize erythritol. *P. tsukubaensis* KN75 produces 245 g/L of erythritol from glucose, corresponding to 2.86 g/L/h productivity and 61% yield [42]. The industrial potential of *P. tsukubaensis* KN75 was also demonstrated at pilot (300 L) and industrial (50,000 L) scales. In addition, food-grade osmophilic yeast *Moniliella* sp. BCC25224 achieved a erythritol production yield of 0.47 g/g glucose in an optimal culture medium [43]. Hence, erythritol yield can be effectively increased by optimizing the culture medium. These metabolic engineering strategies can be further investigated in natural erythritol producers to develop a more effective microbial platform for erythritol production.

### 3.3. Threitol

Threitol is a four-carbon polyol produced by certain osmotolerant yeasts as an osmoprotective agent. It is widely applied in green chemistry, and in the pharmaceutical and food industries. It is also the precursor of treosulfan, an ovarian cancer drug [67]. Threitol is produced as an antifreeze agent in *Armillaria mellea* and in the Alaskan beetle *Upis ceramboides* to tolerate prolonged freezing to a low mark of −50 °C [16,68]. Threitol is a diastereoisomer of erythritol, and it is formed from erythritose by xylitol dehydrogenase (XDH). The xylitol dehydrogenase gene (Ss-XDH) derived from *S. stipitis* CBS 6054 irreversibly oxidizes erythritol to erythrulose and subsequently reduces erythrulose to threitol [69]. A threitol titer of 112 g/L with a yield of 0.37 g/g total substrate from glucose was observed in *Y. lipolytic* harboring Ss-XDH. However, the overexpression of XDH activated the mannitol synthesis pathway, which increases the intracellular concentration of mannitol to balance the osmotic pressure. To reduce mannitol (by-product) production, the culture supernatant was used as a substrate for the yeast *C. parapsilosis* strain CGMCC2.4312, which consumes mannitol and erythritol, but not threitol. This step led to 98% pure threitol in the fermentation broth [16]. This simple and efficient two-step biological process enabled the high-yield production of high-purity threitol. This work demonstrates the potential of *Y. lipolytica* for the sustainable production of threitol from renewable resources.

## 4. Biosynthesis of Glycosides

Glycosides, which serve as sugar substitutes, are primarily derived from plants with sweet-tasting leaves or fruits. The aglycones of these glycosides are generally terpenoids, as their synthesis occurs predominantly in the endoplasmic reticulum (ER), catalyzed by CYP450s. Three kinds of glycosides—rubusosides, glycyrrhizin, and mogrosides—have been discovered to be sugar substitutes. Even though the in vivo biosynthetic pathways of the glycosides have been elucidated [70,71,72], the de novo synthesis of mogroside in yeast has not been established thus far. Therefore, only the biosynthesis of rubusosides and glycyrrhizin in yeast are discussed in this section.

### 4.1. Rubusosides

Rebaudiosides exhibit superior taste among the steviol glycosides (SGs) extracted from *Stevia rebaudiana* leaves. This novel natural sweetener can be used as a general-purpose sweetener, as it has zero calories and high sweetness (114–350 times higher than sucrose) [3]. Rebaudiosides have attracted significant attention in recent years and have been widely applied in the food and beverage industries [73]. To date, more than 60 different SGs have been isolated from *Stevia rebaudiana*, including rubusoside, stevioside, rebaudioside A (Reb A), rebaudioside B (Reb B), rebaudioside D (Reb D), and rebaudioside M (Reb M) [3]. The United States Food and Drug Administration has been petitioned for the acceptance of Reb A, Reb D, and Reb M as safe (GRAS) [73,74]. Stevioside and Reb A are found in *S. rebaudiana* leaves and used as major ingredients in commercial Stevia sweeteners. However, they have a bitter aftertaste, which limits their applications in food products [75]. Meanwhile, Reb D and Reb M are sweeter than stevioside and Reb A. Thus, they are considered the next-generation ingredients for Stevia sweeteners [76]. However, scaling up traditional extraction approaches is not feasible due to the low SG content (less than 0.1% dry weight) in Stevia leaves [77]. Hence, alternative SG production strategies such as multiple-enzymatic cascade reactions are needed.

**Figure 3:**
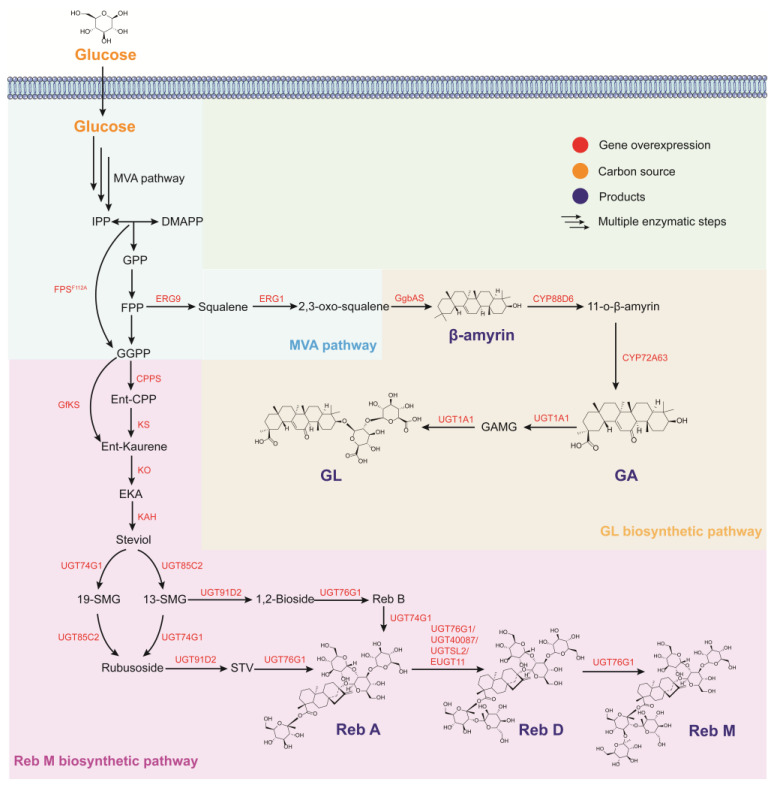
Biosynthetic pathway of steviol glycosides and glycyrrhizin in yeasts. FPS: farnesyl pyrophosphate synthase; CPPS: ent-copalyl diphosphate synthase; KS: kaurene synthase; GfKS: kaurene synthase from *Gibberella fujikuroi*; KO: ent-kaurene oxidase; KAH: kaurenoic acid 13α-hydroxylase; EKA: ent-kaurenoic acid; UGT74G1, UGT85C2, UGT91D2, UGT40087, UGTSL2, EUGT11 and UGT1A1: UDP-glycosyltransferase; GgbAS: β-amyrin synthase; CYP88D6 and CYP72A63: cytochrome P450 enzymes; GA: glycyrrhetinic acid; GAMG: glycyrrhetic acid 3-O-mono-β-*d*-glucuronide; EKA: ent-kaurenoic acid; 19-SMG: steviol-19-O-glucoside; 13-SMG: Steviol-13-O-monoglucoside; STV: stevioside; Reb A: rebaudioside A; Reb B: rebaudioside B; Reb D: rebaudioside D; Reb M: rebaudioside M.

SGs are biosynthesized via the mevalonate (MVA) pathway, where acetyl-CoA produces GGPP, which is cyclized to ent-kaurene by kaurene synthase, followed by oxidation and hydroxylation catalyzed by two P450 enzymes to produce steviol. Finally, SGs with different sugar units are formed via different glycosyltransferases [11]. SGs can be produced by enzymatic and whole-cell catalytic synthesis. Three UGTs (UGT85C2, UGT74G1, and UGT76G1) involved in major glycoside biosynthesis have been isolated from *S. rebaudiana*. UGT85C2 adds glucose to the C-13 hydroxyl of steviol, UGT74G1 adds glucose to the C-19 carboxyl group, and UGT76G1 transfers one sugar to another (Figure 3) [70]. The UGT76G1 was expressed in *S. cerevisiae* with whole-cell reaction parameters such as cell permeability (1% toluen), temperature (30 °C), pH (pH = 7.2), citrate acid (15 g/L), and Mg^2+^ concentrations (6 g/L), as well as glucose feeding optimized (40 g/L), and 1160.5 mg/L of Reb A was produced from 2 g/L of stevioside in 48 h in the absence of extracellular UDP-glucose [73]. In vitro experiments with UGT76G1 yielded Reb M from Reb D with a 72.2% molar conversion [77]. However, the UGT76G1 glycosyltransferase exhibits a broad substrate scope, resulting in the formation of by-products and a low yield of the desired product.

The enzymatic conversions of Reb A to Reb D and Reb D to Reb M require optimization to produce high-purity Reb M. Screening a UGT76G1 mutant library revealed that UGT76G1^Thr146Gly^ and UGT76G1^His155Leu^ could aid in the accumulation of Reb D and Reb M while reducing the accumulation of unwanted by-products [76]. Notably, UGTSL2 from *Solanum lycopersicum* was identified as producing Reb D from Reb A. A multi-enzyme reaction system with UGT76G1, UGTSL2m, and StSUS1 (*Solanum tuberosum* sucrose synthase) exhibited a two-step glycosylation of stevioside to produce Reb D. However, Reb M2 is a by-product of this reaction. Subsequently, a UGTSL2^Asn358Phe^ mutant achieved Reb D accumulation from 84.4% to 92.5%, and the Reb M2 content decreased from 3.7% to 0.4% [74]. Another study employed a two-step temperature-control strategy with EUGT11 derived from *Oryza sativa* to produce Reb D in *Pichia pastoris*, reaching a 95.31% conversion rate at 28/35 °C compared to a conversion rate of 62.41% in a one-step process at 28 °C [78]. This strategy simplifies the procedure and reduces the cost to achieve highly efficient production of Reb D. 

Unlike pure enzyme reactions or whole-cell reaction systems, microbial cell factories are limited in the case of SG production because of the rate-limiting steps involving CYP450s; the accumulation of intermediate metabolites leads to cytotoxicity or growing pressure, as well as the imbalance of the metabolic network [3,79]. Recently, a study used an engineered *S. cerevisiae* strain to produce 1368.6 mg/L and 132.7 mg/L of rubusoside and rebaudiosides (Reb A, Reb D, and Reb M), respectively, from glucose in 15 L bioreactors [11]. The corresponding engineered yeast strain can be adopted to achieve continuous large-scale production of asparagus glycosides and their derivatives. However, the weak catalytic activity of CYP450s and the poor substrate specificity of UGTs are the main challenges for SGs production in microbial cell factories. Therefore, it is necessary to enhance the catalytic activity of these enzymes, especially CYP450s and UGT76G1.

### 4.2. Glycyrrhizin (GL)

Glycyrrhizin or glycyrrhizic acid (GL) is a terpenoid derived from glycyrrhetinic acid (GA), 170 times sweeter than sucrose, and calorie-free [80,81]. GL also has potential applications as an anti-asthma and anti-inflammatory agent as well as a neuroprotectant [81]. It is extracted from the plant *Glycyrrhiza uralensis*, where the separation of GL from similar compounds such as GA and Glycyrrhetic Acid 3-O-mono-β-*d*-glucuronide (GAMG) is difficult. Hence, developing efficient biosynthetic routes to produce GL is essential.

GL is biosynthesized from the MVA pathway to form the triterpenoid 2,3-oxidosqualene as the precursor for downstream transformations. 2,3-Oxidosqualene is converted to β-amyrin in one step, and the C11 and C30 of β-amyrin are oxidized by two CYP450s to produce GA. Two molecules of glucuronic acid are introduced at the 3-OH of GA with an α-(1→1)-glycosidic bond. UGTs catalyze the stepwise reaction between UDP-glucuronic acid and GA (Figure 3). β-Amyrin, which has a triterpenoid skeleton, has been previously produced in *S. cerevisiae* [82]. However, the biosynthesis of GL is restricted by the inefficient heterologous expression of CYP450s and UGTs. Hence, mining feasible CYP450s and UGTs is essential for optimizing the abovementioned biosynthetic routes.

CYP88D6 and CYP72A154 were isolated from *G. uralensis* using transcriptome sequencing and analyzing, but the production titer (15 μg/L and 76 μg/L) was too low to apply in industrial scale production because of low efficiency [83]. Two strategies were considered to improve the yield of GL: (i) mining highly efficient CYP450s from other sources and (ii) introducing a complete oxidation-reduction system, namely cytochrome P450 reductases (CPRs), to balance the electronic transformations [84]. CYP88D6 and CYP72A154 were replaced by Uni25647 and CYP72A63, respectively, which were identified from an online database, and *G. uralensis* CPR1 was introduced as the reduction system. The 5L fed-batch fermentation of the engineered strain afforded 18.9 mg/L of GA. In the case of mining new UGTs, some UGTs in *G. uralensis* transform GA to GAMG [85], or GAMG to GL [86,87], but these UGTs have not been expressed in yeast chassis. UGT1A1 was the best UGT identified from the *Homo sapiens* UGTs library that could convert GA to GAMG and GL in *S. cerevisiae*, which yielded a GL titer of 5.98 mg/L [88]. However, the yield is insufficient for massive production. Therefore, enhancing the activities of related CYP450s and UGTs is crucial, as well as increasing the strain tolerance of GL, as it may be toxic to cell growth [44].

## 5. Biosynthesis of Rare Monosaccharide

Rare monosaccharides, also known as rare sugars, are less-abundant natural monosaccharides. Twenty hexoses and nine pentoses have been identified as rare monosaccharides thus far. *l*-arabinose, *d*-fructose, *d*-galactose, *d*-glucose, *d*-mannose, *d*-ribose, and *d*-xylose are common monosaccharides [89]. The rare monosaccharides d-psicose and *d*-tagatose have attracted attention as sugar substitutes, as they are as sweet as sucrose but have a lower energy content than sucrose and can be used in healthcare products [3].

### 5.1. d-Psicose

*d*-Psicose, also known as *d*-allulose, is a ketohexose mainly found in wheat and cane molasses. *d*-psicose and *d*-fructose are epimers with different conformations at 3-OH. The sweetness of *d*-psicose is 0.7 times that of sucrose, and it is nearly calorie-free. Moreover, it can regulate blood sugar and blood fat levels by modulating energy intake [90]. The conventional extraction and chemical synthesis of *d*-psicose are difficult to scale up due to high costs of substrates and equipment and the complex operation of separation from other similar monosaccharides.

**Figure 4:**
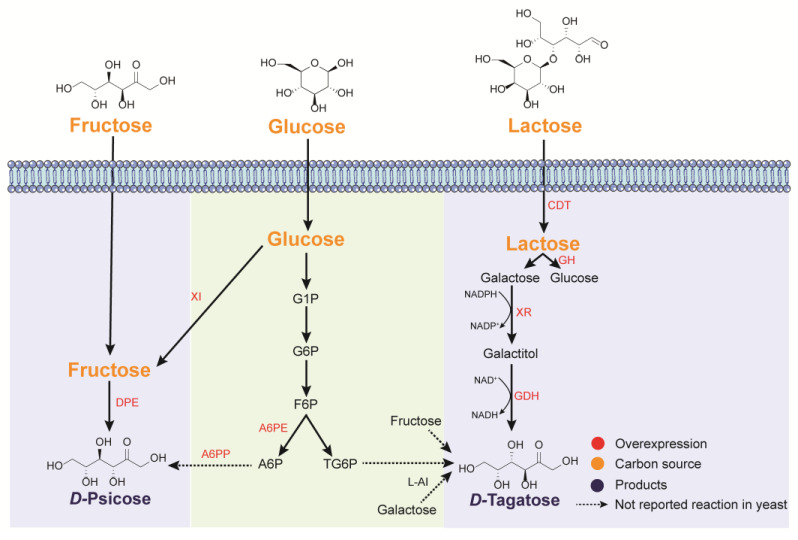
Enzymatic reactions and biosynthetic pathways for the formation of rare monosaccharides in yeasts. The synthetic pathways with the green background have not been reported in yeasts but have been realized in enzymatic reactions. XI: xylose isomerase; CDT: cellobiose transporter; GH: β-glucosidase; XR: xylose reductase; GDH: galactitol dehydrogenase; *l*-AI: *l*-arabinose isomerase; DPE: *d*-psicose 3-epimerase; A6PE: d-allulose 6-phosphate epimerase; A6PP: d-allulose 6-phosphate phosphatase; A6P: psicose 6-phosphate; TG6P: tagatose 6-phosphate.

Two biosynthetic pathways exist for *d*-psicose formation (Figure 4). The key step in the first pathway is the epimerization of *d*-fructose by *d*-psicose 3-epimerase (DPEs). Thus, DPEs have been identified from more than ten different sources and characterized in vitro for the conversion of *d*-fructose to *d*-psicose [90]. The second pathway for *d*-psicose formation is the in vivo phosphorylation of *d*-fructose 6-phosphate (F6P) to *d*-psicose 6-phosphate (A6P) by *d*-allulose 6-phosphate epimerase (A6PE). A6P is then converted to *d*-psicose by *d*-allulose 6-phosphate phosphatase (A6PP) [91]. Moreover, *d*-psicose could be generated via other reactions with cheaper carbon sources such as *d*-glucose, starch, and maltodextrin [92,93].

To date, the de novo production of *d*-psicose in yeast has not been reported. However, a one-step whole-cell transformation of *d*-psicose has been realized via the overexpression of DPEs in yeast cells [45]. However, the optimal temperature of DPEs is higher than the maximum growth temperature of most yeast species. *Kluvyeromyces marxianus* shows excellent thermotolerance [94]. Therefore, a DPE isolated from *Agrobacterium tumefaciens* was overexpressed in a screened *K. marxianus* strain, and 190 g/L of *d*-psicose using 750 g/L of *d*-fructose at 55 °C in 12 h was produced [45]. Still, the cost of *d*-fructose is high. Thus, *d*-xylose isomerases (XIs) were employed to enable the utilization of a cheaper carbon source, *d*-xylose. The co-expression of DPE and XI in *S. cerevisiae* spores increased the yield of *d*-psicose to 12.0% [95]. However, none of these methods meet the requirements of industrial production. In the future, protein engineering on DPEs or mining other pathways to increase conversion rate will be necessary. Moreover, new strategies for *d*-psicose separation are important as well.

### 5.2. d-Tagatose

*d*-Tagatose is also a ketohexose like *d*-psicose. However, it is an epimer of *d*-fructose with a different conformation at 4-OH. *d*-Tagatose has ~90% of the sweetness of sucrose but only a quarter of the energy content of sucrose. Both *d*-tagatose and *d*-psicose are reducing sugars and can undergo the Maillard reaction at high temperatures. In other words, these sugars are suitable sugar substitutes for cooking and baking [96]. *d*-Tagatose is also found in dairy products (yogurt, cheese, milk powder, etc.), which suggests that its biosynthesis is related to *d*-galactose or lactose in dairy products [97].

*d*-Tagatose has two different biosynthetic routes, based on epimerization and oxidoreduction, respectively (Figure 4). Many *d*-tagatose production routes involve epimerization, including the formation of cheap dairy by-products (cheese whey powder, etc.) or the upstream intermediate disaccharide lactose [98]. *l*-arabinose isomerases (*l*-AIs) can convert *d*-galactose to *d*-tagatose. β-*d*-tagatose galactosidase and *l*-AI were co-expressed in *P. pastoris* to produce *d*-tagatose from lactose, and a 30% conversion was achieved, which was higher than that of most in vitro enzymatic reactions [99]. *d*-tagatose can also be produced from the epimerization of *d*-fructose. However, enzymes with excellent catalytic activity for *d*-fructose 4-epimerization are rare, even though numerous UDP-hexose 4-epimerases have been identified in various organisms [100,101]. Thus, researchers engineered other enzymes to change their substrate selectivity. For example, *d*-tagaturonate 3-epimerase UxaE was engineered to produce *d*-tagatose [102]. The enzymatic transformation catalyzed by the mutant could achieve 213 g/L *d*-tagatose from 700 g/L *d*-fructose. Moreover, an alternative route for *d*-tagatose production is the conversion of *d*-fructose 6-phosphate to *d*-tagatose 6-phosphate by aldolases. *d*-tagatose 1,6-bisphosphate aldolase GatZ from *Caldilinea aerophila* was introduced into *E. coli* to produce *d*-tagatose using the cheap substrate maltodextrin [103]. These results demonstrated the feasibility of producing *d*-tagatose by the epimerization pathway. Moreover, the epimerization pathway may be applied in thermophilic yeasts such as *Kluyveromyces marxianus*. Additionally, the chemical equilibrium of the epimerization reaction limits further optimization of the desired conversion. Therefore, the one-step epimerization has been replaced by a two-step oxidoreduction process. In this oxidoreduction pathway, *d*-galactose is converted to galactitol and then to *d*-tagatose catalyzed by *d*-xylose-1-reductase isolated from *S. stipites* (*XYL1*) and galactitol-2-dehydrogenase from *Rhizobium leguminosarum* (*GDH*), respectively. Moreover, this *S. cerevisiae* chassis was engineered to use cellobiose or lactose as its only carbon source. The final titer of *d*-tagatose was 37.69 g/L (2 L fed-batch, 300 h) and the yield of *d*-tagatose from lactose was 0.33 g/g, with a 9:1 ratio of *d*-tagatose and *d*-galactose under optimized conditions [32]. In this route, *d*-galactose was efficiently transformed into *d*-tagatose rather than being limited by equilibrium. However, further optimization is required. For instance, transporting the intermediate galactitol out of the cell might decrease the generation of *d*-tagatose. Thus, genetic perturbations of monosaccharide or sugar alcohol transporters could be considered, such as aquaglyceroporin encoded by Fps1.

## 6. Conclusions and Perspectives

Natural sugar substitutes have attracted significant attention, as they are safe and low in calories, and exhibit various physiological functions. In recent decades, the biosynthesis of natural sugar substitutes in engineered yeast strains has emerged as an alternative to current chemical manufacturing methods. In this review article, we have described the recent advances in yeast biosynthesis of natural sugar substitutes. To date, only erythritol has been produced at an industrial scale [42], although the biosynthesis of other natural sugar substitutes should be optimized through further engineering. In this context, the commercialization of biosynthesized natural sugar substitutes has not been fully achieved, as our collective understanding of natural plant enzymes is limited, and dynamic metabolic regulation strategies need to be developed.

The formation of by-products in the biosynthesis process is a common issue, particularly for those processes with renewable substrates [16,36]. Even though strategies to engineer the yeast genomes to reduce the formation of by-products have been reported, knowledge of specific biosynthetic genes and their regulations is limited. For example, the expression of a heterologous dehydrogenase may promote the biosynthesis of sugar alcohols; it also generates by-products, and only a few mannitol dehydrogenases have been identified so far. Thus, understanding the regulation of related genes not only reduces by-product formation but also helps to develop appropriate engineering strategies for sugar alcohol biosynthesis.

In addition, the expression levels or the substrate spectra of heterologous key enzymes are usually not satisfactory. Although *S. cerevisiae* is considered to be a superior host for expressing plant-derived P450 [104], low expression levels or catalytic activities are often observed, such as in the cases of Uni25647 and CYP72A63 for glycyrrhizin synthesis, as well as KO and KAH for rubusoside synthesis [11,88]. Fusion protein engineering strategy is often employed to enhance the catalytic efficiency of P450 enzymes. The production level, production rate, and overall production ratio of these fusion proteins are improved by optimizing the length of the linker and the orientation of the active sites. Additionally, artificial intelligence tools, such as ec_iML1515 [105] and MaSIF [106], can be used to design catalytically active pockets to improve the catalytic activity and substrate specificity of the corresponding enzymes in yeasts. The recent rapid development of metabolic engineering, protein engineering, synthetic biology, and artificial intelligence technology could promote the advancement of the biosynthesis of natural sugar substitutes in the near future.

## Figures and Tables

**Table 1:** Summary of natural sugar substitutes’ production in yeasts.

Types	Products	Hosts	Substrate	Fermentation Condition	Titers (g/L)	Yield (g/g)	Productivity (g/L/h)	Reference
Oligosaccharides	2′-Fucosyllactose	*S. cerevisiae*	Fucose and Lactose	Shake Flask	0.503	NA ^1^	NA	[22]
		*S. cerevisiae*	Xylose and Lactose	Fed-Batch Bioreactor	25.5	NA	0.35	[29]
		*S. cerevisiae*	Glucose and Lactose	5 L Fed-Batch Bioreactor	26.63	NA	0.28	[30]
		*S. cerevisiae*	Glucose and Lactose	5 L Fed-Batch Bioreactor	32.05	NA	0.67	[31]
	Trehalose	*Y. lipolytica*	Maltose	3 L Batch Bioreactor	219	0.73	4.5	[34]
Sugar alcohols	Mannitol	*S. cerevisiae*	Glucose	Shake Flask	NA	0.12	NA	[35]
		*C. magnoliae*	Glucose	2.5 L Fed-Batch Bioreactor	240	0.81	4	[36]
		*C. parapsilosis*	Glucose	30 L Fed-Batch Bioreactor	97.1	0.34	0.81	[37]
		*C. magnoliae*	Glucose	10 L Fed-Batch Bioreactor	223	0.89	1.72	[38]
		*Y. lipolytica*	Glucose	5 L Fed-Batch Bioreactor	98.2	0.33	1.1	[14]
	Erythritol	*Y. lipolytica*	Glycerol	5 L Fed-batch Bioreactor	220	0.43	0.54	[39]
		*Y. lipolytica*	Glucose	Bench-Top Reactors	148	NA	NA	[40]
Sugar alcohols	Erythritol	*Y. lipolytica*	Glucose	30 m^3^ Fed-Batch Bioreactor	196	0.65	2.51	[41]
		*Y. lipolytica*	Crude glycerol	5 L Fed-Batch Bioreactor	150	0.62	1.25	[15]
		*P. tsukubaensis*	Glucose	5 L Fed-Batch Bioreactor	245	0.61	2.86	[42]
		*Moniliella*	Glucose and soybean flour	10 L Fed-batch Bioreactor	86.6	0.47	0.40	[43]
	Threitol	*Y. lipolytica*	Glucose	Shake Flask	112	0.37	NA	[16]
Glycosides	Rubusoside	*S. cerevisiae*	Glucose	15 L Fed-Batch Bioreactor	1.37	NA	NA	[11]
	Reb A	*S. cerevisiae*	Glucose	15 L Fed-Batch Bioreactor	21.5 mg/L	NA	NA	[11]
	Reb D	*S. cerevisiae*	Glucose	15 L Fed-Batch Bioreactor	44.2 mg/L	NA	NA	[11]
	Reb M	*S. cerevisiae*	Glucose	15 L Fed-Batch Bioreactor	67.0 mg/L	NA	NA	[11]
	GL	*S. cerevisiae*	Glucose	Shake Flask	5.98 mg/L	NA	NA	[44]
Rare monosaccharide	*d*-Psicose	*K. marxianus*	Fructose	Whole-cell Reaction	190	0.253	15.83	[45]
	*d*-Tagatose	*S. cerevisiae*	Lactose	2 L Fed-Batch Bioreactor	37.69	0.526	0.1126	[32]

^1^ NA: Not available.

## Data Availability

Data sharing is not applicable to this article, as no datasets were generated or analyzed during the current study.
